# Automated Detection of Epileptic Seizures in EEG Signals via Micro-Capsule Networks

**DOI:** 10.3390/brainsci15080842

**Published:** 2025-08-07

**Authors:** Baozeng Wang, Jiayue Zhou, Hualiang Zhang, Jin Zhou, Changyong Wang

**Affiliations:** 1 Beijing Institute of Basic Medical Sciences, Beijing 100850, China; baoer723166@163.com (B.W.); liangzai951@sohu.com (H.Z.); 2 Beijing Shijitan Hospital, Capital Medical University, Beijing 100038, China; zhoujiayue1016@163.com; 3 Chinese Institue for Brain Research, Beijing 102206, China

**Keywords:** epileptic, automatic detection, micro-capsule networks, electroencephalogram (EEG), classification performance

## Abstract

Background: Epilepsy is a chronic neurological disorder that affects individuals across all age groups. Early detection and intervention are crucial for minimizing both physical and psychological distress. However, the unpredictable nature of seizures presents considerable challenges for timely detection and accurate diagnosis. Method: To address the challenge of low recognition accuracy in small-sample, single-channel epileptic electroencephalogram (EEG) signals, this study proposes an automated seizure detection method using a micro-capsule network. First, we propose a dimensionality-increasing transformation technique for single-channel EEG signals to meet the network’s input requirements. Second, a streamlined micro-capsule network is designed by optimizing and simplifying the framework’s architecture. Finally, EEG features are encoded as feature vectors to better represent spatial hierarchical relationships between seizure patterns, enhancing the framework’s adaptability and improving detection accuracy. Result: Compared to existing EEG-based detection methods, our approach achieves higher accuracy on small-sample datasets while maintaining a reduction in computational complexity. Conclusions: By leveraging its micro-capsule network architecture, the framework demonstrates superior classification accuracy when analyzing single-channel epileptiform EEG signals, significantly outperforming both convolutional neural network-based implementations and established machine learning methodologies.

## 1. Introduction

Epilepsy, a chronic neurological disorder affecting individuals of all ages, impacts an estimated 50 million people worldwide [1]. It is characterized by sudden or rhythmic abnormal neuronal discharges that can cause brain injury [2], leading to loss of consciousness, intense localized or generalized muscle contractions, and even life-threatening conditions [3]. Therefore, early detection of epilepsy in patients is crucial to prevent the spread of epileptic lesions in the brain [4].

Due to the potential adverse effects of antiepileptic drugs on the nervous system [5], electroencephalogram (EEG) monitoring provides an important non-pharmacological solution for real-time detection and intervention of epileptic seizures, while avoiding drug-related complications [6]. Based on altered neuronal activation patterns, EEG signals in epileptic patients are categorized into four states: pre-ictal, ictal, post-ictal, and inter-ictal [7]. The first three stages exhibit distinct differences in EEG signal amplitude and frequency, providing a basis for seizure detection [8]. However, manual EEG analysis by neurologists is time-consuming, requiring extensive observation of signals. Additionally, the process is labor-intensive and highly dependent on the clinician’s expertise, increasing diagnostic burdens [9]. Thus, there is a critical need for automated seizure detection systems that enable real-time monitoring and alerts via computer-aided technologies [10].

Modern machine learning and deep learning techniques have become pivotal tools for seizure detection, driven by advances in computer-aided systems [11]. Prior research demonstrates that optimizing EEG features prior to classification is critical for improving seizure detection accuracy [12,13,14]. EEG feature extraction approaches primarily encompass two categories: manual extraction using classical algorithms and automated extraction via deep learning [15]. Classic artificial methods construct features by analyzing the time-domain (such as statistical moments: mean, variance, kurtosis; nonlinear features: entropy, Lyapunov exponent), frequency-domain (such as power spectral density), and time-frequency domain (such as wavelet transform) characteristics of signals. In addition, dimensionality reduction techniques (such as principal component analysis) and spatial filtering methods (such as common spatial pattern, often combined with wavelet transform) are also commonly used to optimize feature expression [16]. The automatic feature methods based on deep learning mainly rely on deep neural networks (such as convolutional neural networks (CNNs), recurrent neural networks (RNNs)) to directly learn hierarchical feature representations from raw or preprocessed EEG signals, reducing reliance on manually designed features [17,18]. However, due to the non-stationary nature of EEG signals, this poses significant challenges for using the above two methods to stably and reliably extract highly discriminative features. This challenge is particularly prominent in applications such as epilepsy detection that require extremely high feature sensitivity.

Following EEG feature extraction, the next step is classifying epileptic EEG signals. Researchers have employed both traditional machine learning and deep learning algorithms for this task [19,20]. Common machine learning classifiers include k-means clustering, support vector machines (SVMs), linear discriminant analysis (LDA), Naive Bayes, Gaussian mixture models, and random forests. However, the performance of those methods largely relies on EEG signal detection tasks with distinctly identifiable features, severely constraining their applicability and generalization across diverse scenarios [21]. Consequently, enhancing models’ capability to detect epilepsy from suboptimal EEG signals becomes paramount to address these challenges. Deep learning approaches include CNN, RNNs, long short-term memory (LSTM) networks, and capsule networks. The core advantage of deep learning algorithms lies in the direct mapping from raw data to classification results, simplifying the separation steps of feature extraction and classification in traditional methods, and enhancing the adaptability of algorithms to different data distributions [22]. However, deep learning models often lack spatial reasoning capabilities (e.g., object orientation) [23,24] and are computationally intensive due to their large-scale architectures, which may include hundreds of layers [25], the training and inference process is usually computationally intensive and requires high hardware resources.

A persistent challenge is the significant distortion of weak EEG signals [26]. Addressing these limitations, Hinton introduced a capsule network framework with vector-based neurons that mimics human cognitive processes and better simulates hierarchical knowledge representation in neural networks [27]. Numerous studies demonstrate the application of capsule networks across diverse domains. Kwon-Woo Ha and Jin-Woo Jeong developed a capsule network framework for classifying motor imagery tasks [28]. Zhang, H. et al. introduced a capsule network architecture to decode motion direction via spike timing analysis [23]. Suat Toraman designed a 1D capsule network for epilepsy classification during seizure and pre-ictal phases [29]. However, capsule networks are rarely applied to multi-class classification tasks in small EEG datasets [30]. The need for specialized personnel (1∼2 professionals), combined with high costs (e.g., single-use EEG caps), elevated scalp-electrode impedance, and time constraints, makes it difficult to collect large-scale epileptic EEG datasets from individual subjects [31]. Thus, small sample sizes remain a significant barrier in practice, and accurate seizure identification from limited EEG data remains an unresolved challenge.

We propose an epilepsy detection method based on capsule neural networks. Architecturally, the capsule network consists of many small, interconnected processing units that encode the presence, orientation, location, and other attributes of objects through capsules. Dynamic routing is employed to propagate information and better preserve spatial hierarchical relationships, thereby enabling the autonomous extraction of spatial relationships from EEG signals and the preservation of key features to improve detection accuracy. The lightweight architecture of this framework enables multi-level feature learning from EEG signals. By utilizing dynamic routing algorithms, the capsule network layer is simplified, which can enhance the accuracy of multi-class classification even with limited EEG samples.

## 2. Methods

First, the EEG datasets and preprocessing steps are described, forming the foundation for subsequent research. Second, the micro-capsule network model is designed, its working mechanism is explained, and the core algorithm workflow of the framework is detailed. Finally, the evaluation metrics for the EEG detection algorithm are defined.

### 2.1. EEG Preprocessing and Generation

In this section, we validate the proposed epilepsy detection framework using the publicly available Bonn EEG dataset. The following section details the EEG datasets, preprocessing steps, and data matrix generation methodology.

#### 2.1.1. Data Sets Description

The dataset is publicly accessible via the EEG time series repository at the University of Bonn, Germany [32]. All EEG recordings utilized a 128-channel amplifier system with a common average reference. Signals were sampled at 173.61 Hz and digitized at 12-bit resolution. Interference components (e.g., muscle activity) were eliminated from the signal using a filter with a bandpass range of 0.53 to 40 Hz and a slope of 12 dB/oct.

The EEG data were segmented into epilepsy-specific stages from continuous recordings. The datasets (labeled A∼E) represent five distinct EEG categories, each containing 100 single-channel EEG files spanning 23.6 s. Each file comprises 4096 data points of an EEG time series encoded in ASCII format. Scalp EEG datasets (A/B; *n* = 5 healthy subjects) cover eye-open/closed states. Intracranial EEG datasets (C-E; *n* = 5 epilepsy patients) include: (i) Interictal recordings: Dataset D (epileptogenic focus) and Dataset C (contralateral hippocampal formation); (ii) Ictal recordings: Dataset E (seizure onset zone).

#### 2.1.2. Data Preprocessing

Since deep learning requires relatively large datasets, limited data can lead to severe overfitting. Typically, overfitting in neural network models is mitigated by expanding the dataset size to leverage prior knowledge. To address this, EEG data are segmented using a fixed sliding window of size *m* (set to 1 s) and overlap $\delta_{m}$ (set to 0∼1), thereby augmenting the training dataset.

Using a sliding window *m* (1 s) and overlap $\delta_{m}$ (0), each 23.6-s EEG file is sequentially segmented. The segmented data are shuffled randomly before training. From the five EEG datasets, 11,500 samples are obtained, each containing 178 data points and 1 class attribute. To combat statistical uncertainty from small-sample datasets during testing, EEG data were first partitioned into training and testing sets at a 7:3 ratio. Then a three-fold cross-validation procedure is employed to evaluate the performance of the epilepsy detection algorithm. Compared to seizure detection models using other deep learning networks trained on tens of thousands of samples [33], this study utilizes small-scale datasets.

#### 2.1.3. Data Matrix Generation

Given the single-channel time-series characteristics of epileptic EEG signals, we retain the time-series EEG signals as input for the micro-capsule network. Additionally, there is no need for channel selection, which usually reduces the amount of computation in the subsequent classification process. To better extract discriminative EEG features and adapt to the micro-capsule network’s architecture, a dimensionality expansion technique transforms the EEG signals into a matrix. The data vectors are reshaped into a three-dimensional structure, with dimensions corresponding to length, height, and the number of EEG feature channels. The EEG data form the matrix elements in each trial, structured as follows:

(1) $$A_{m\times n}=\left[\begin{array}{cccc} a_{11} & a_{12} & \ldots & a_{1n}\\ a_{21} & a_{22} & \ldots & a_{2n}\\ \vdots & \vdots & \ddots & \vdots \\ a_{m1} & a_{m2} & \ldots & a_{mn} \end{array}\right]$$

where $A_{m\times n}$ is a three-dimensional matrix composed of one piece of sample data, *m* is the sampling point number of fragments analyzed in a sample, and *n* is the number of analysis segments in a sample.

### 2.2. Construction of the Micro-Capsule Networks

To enhance the decoding efficiency of individual EEG samples, this study proposes a compact capsule network-based epilepsy recognition model. The framework integrates features of epileptic EEG signals with a dynamic routing mechanism, optimizing both signal characteristics and algorithmic adaptability.

#### 2.2.1. The Micro-Capsule Network Model Establishment

**Figure 1:**
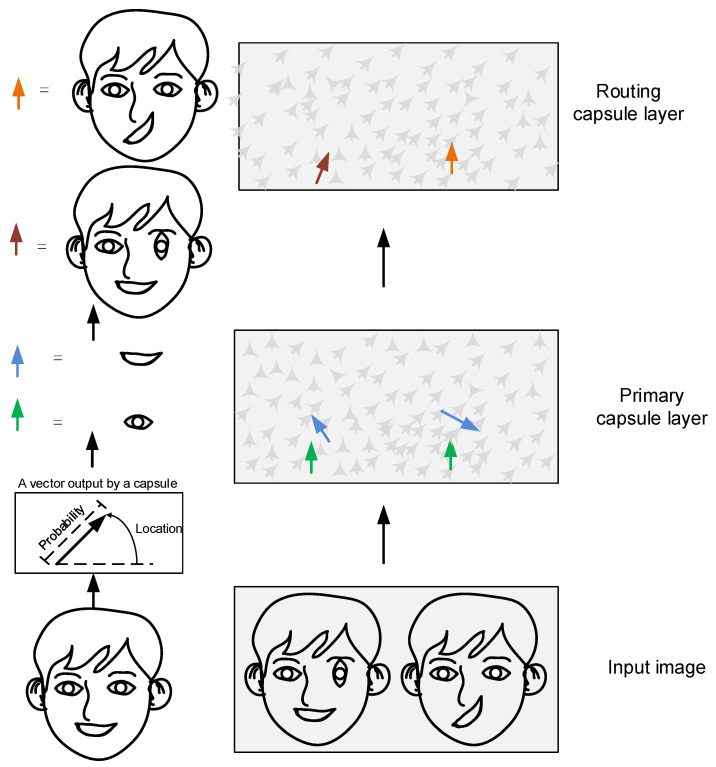
Schematic diagram of automatic encoding for micro-capsule networks. The light green and blue arrows represent the pose and rotation vectors of the eyes and mouth, respectively. The deep red and orange arrows represent the pose of the head sculpture with misaligned right eye and mouth.

To effectively identify dynamic image targets, capsule neural networks preserve spatial relationships between objects. These networks comprise multiple neuron nodes, each encoding instantiation parameters such as pose (position, size, orientation), deformation, velocity, hue, and texture. For example, consider two facial features (the mouth and eyes) to illustrate spatial relationships in detail, as shown in Figure 1.

As shown in Figure 1, after image data is input into the capsule neural network model, neurons in the primary capsule layer learn to detect visual features (e.g., mouth and eyes) and generate vectors with larger magnitudes in regions matching the target features. These vectors encode the contour, shape, and spatial orientation of detected features. Conversely, smaller vectors with randomized directional patterns are produced in non-matching regions.

The routing algorithm assesses recognition accuracy for the target graph and its sub-graphs. Output data are directed to capsule structures representing the mouth and eyes. When the eye sub-graph is processed via routing, the mouth features exhibit poor correlation with the target characteristics, reducing their routing weight. Following the mechanism where low-level capsules assess high-level capsules to enhance output receptivity, improved feature alignment with the eye sub-graph increases the routing weight. This prioritizes input signals to the eye capsule structure, while the mouth capsule structure captures fewer relevant signals.

Assume that the input vectors ($u^{1}$, $u^{2}$ and $u^{3}$) received by the capsule originate from three lower-layer capsules. The vector’s length represents the probability of features detected by the lower-layer capsules, while its direction encodes the spatial state of detected features (e.g., position or orientation).

Assume the lower capsule detects specific local features (e.g., eyes, mouth, and nose), while the output capsule recognizes the composite structure (e.g., a face). The vector is multiplied by its corresponding weight matrix $W_{\mathrm{ij}}$, which captures spatial and hierarchical connections between low-level features (eyes, mouth, nose) and high-level features (face). This transformation predicts the spatial properties of the high-level features, such as position, orientation, and scale.

The reconstruction loss method is typically employed to optimize weight parameters within the capsule hierarchy, replicating autoencoder-like functionality. By leveraging this approach, capsule networks explicitly model hierarchical feature relationships. Capsule neurons utilize unique parameters to instantiate specific categories (e.g., objects in an image), with their output values representing the probability of a target’s presence, thereby enhancing detection accuracy. Moreover, unlike CNNs, which require large volumes of training data, capsule networks achieve state-of-the-art performance with minimal samples.

#### 2.2.2. Epilepsy Detection Based on the Micro-Capsule Network Model

**Figure 2:**
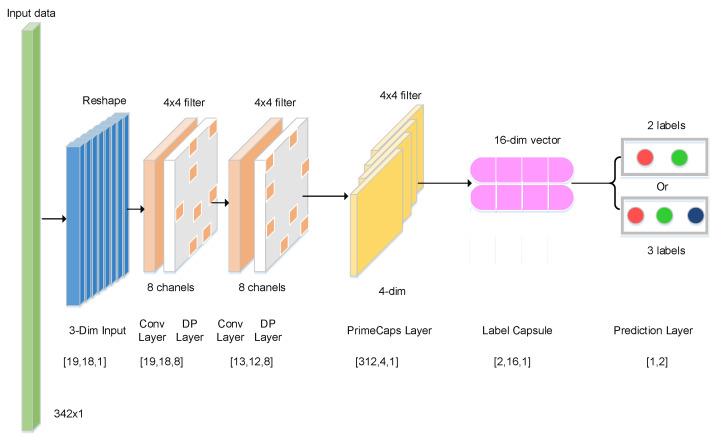
The number of network layer and structural parameters for micro-capsule network architecture.

Targeting small-sample epilepsy EEG datasets, we propose a micro-capsule network architecture tailored for limited-data datasets. Reconstructing epileptic EEG signals exerts minimal influence on recognition accuracy. Excessively deep networks introduce excessive parameters, reducing training speed. To simplify the micro-capsule network and improve training efficiency, we omit the reconstruction layer. The capsule network’s fully connected layers employ techniques like sparse connectivity and parameter sharing, significantly decreasing trainable parameters. Taking binary classification as an example, Figure 2 illustrates the micro-capsule network architecture, comprising convolutional, capsule, fully connected, and prediction layers.

The proposed model employs a capsule network architecture to design an end-to-end network capable of effectively distinguishing different epileptic EEG signals. Taking binary classification (AB vs. E) as an example, the number of network layers and structural parameters of the micro-capsule architecture is detailed in Table 1.

The layer architecture and structural parameters of the micro-capsule network are configured as follows. First, the 1D EEG input undergoes dimensionality expansion, transforming into a 3D matrix of shape (19,18,1). This matrix is processed by the initial convolutional layer to generate primary features (19,18,8). Subsequently, a second convolutional layer extracts abstract features (13,12,8). These features are then routed to the primary capsule layer, producing high-dimensional capsule outputs (312,4,1). Next, the deep feature vector is passed through the fully connected label capsule layer, where it is multiplied by a weight matrix to derive a refined eigenvector (2,16,1). Finally, the prediction layer converts these features into classification probabilities either (2,1) for binary tasks or (3,1) for ternary classification enabling epileptic EEG signal identification.

#### 2.2.3. Margin Loss of the Micro-Capsule Network Model

For detecting multiple classes of epileptic EEG signals, margin loss values for each class are computed via dedicated margin loss functions, and the total loss value is derived through summation. After the trained model is obtained [34], the margin loss function $L_{k}$ can be computed with Equation (2), which determines if an object of a particular class exists.

(2) $$L_{k}=p_{k}\max{\left(0,m^{+}-∥v_{k}∥\right)}^{2}+\lambda \left(1-p_{k}\right)\max{\left(0,∥v_{k}∥-m^{-}\right)}^{2}$$

where $p_{k}$ is epileptic classification (binary or three classifications). $∥v_{k}∥$ is the length of the vector *k*. λ is used to adjust the error ratio of different classes, and the value of λ is set to 0.5. The hyperparameters of $m^{+}$ and $m^{-}$ are upper and lower boundaries set to 0.9 and 0.1, respectively.

#### 2.2.4. Integrated Optimization Algorithm of Micro-Capsule Networks

To enhance the detection accuracy of epileptic EEG signals using the micro-capsule network model, these signals are designated as low-level routing nodes, while the overall objective is designated as a high-level routing node. After optimizing the weights, the model links the low-level routing nodes to their corresponding high-level routing nodes [27]. This process is implemented through a dynamic routing protocol.

To improve the epileptic recognition rate, the proposed model can further optimize and detect different epileptic tasks through a dynamic routing protocol. The corresponding parameters and formulas are detailed in the literature [27,35], and the routing algorithm from the following aspects:

(1) The input EEG eigenvector *u* is multiplied by a different weight matrix *W* to generate the output of capsule networks $U_{j|i}$; (2) calculate all coupling coefficients $C_{ij}$ based on softmax function; (3) the EEG eigenvector $U_{j|i}$ is multiplied by coupling coefficient $C_{ij}$ to obtain the weighted vector. The vector $s_{j}$ can be obtained by summing weighted vectors of all capsule neurons in the next layer; (4) the square compression activation function is used to compress weighted sum $s_{j}$ to obtain updated capsule neural unit; (5) after several iterations, the output vector of high-level capsule networks can be calculated and the corresponding weight $B_{ij}$ can be updated;(6) after weight $B_{ij}$ is updated, the algorithm returns to start calculation $C_{ij}$ and repeats *r* times until the training model is obtained; (7) the individual margin loss function $L_{k}$ is used to calculate the margin loss value of each category, and total loss value Loss is obtained by the cumulative method. If the total loss value Loss is less than threshold $L_{th}$, the output layer corresponds to the detection of epileptic seizures. Otherwise, the algorithm continues to optimize the weight coefficient *W*.

### 2.3. Evaluation Metrics

To evaluate the performance of the epileptic detection model, cross-validation can reduce overfitting to a certain extent and obtain as much adequate information as possible under a small sample size. Three evaluation metrics (accuracy, sensitivity and specificity) were used to evaluate the model’s performance. Accuracy predicts correct results as a percentage of the total sample. Sensitivity represents the proportion of true positive samples in actual positive samples. Specificity represents the proportion of true negative samples in actual negative samples.

To better fit the proposed epileptic detection model, the epileptic dataset is stored in a text file, and the training and test datasets are read through the panda’s library and stored in a data frame data structure. This model’s training and testing processes were tested on a windows desktop with 32 GB of RAM and an NVIDIA TITAN RTX GPU (NVIDIA Corporation, Santa Clara, CA, USA) using the Python 3.8.8 environment and TensorFlow-GPU 2.3.0 for good computational efficiency.

## 3. Results

To evaluate the efficacy of the micro-capsule network model in epileptic signal recognition, this study assesses its capabilities across three metrics: the number of network iterations, the recognition rate across different time intervals, and its performance in epileptic detection.

### 3.1. Effect of Iterations on the Micro-Capsule Networks

**Figure 3:**
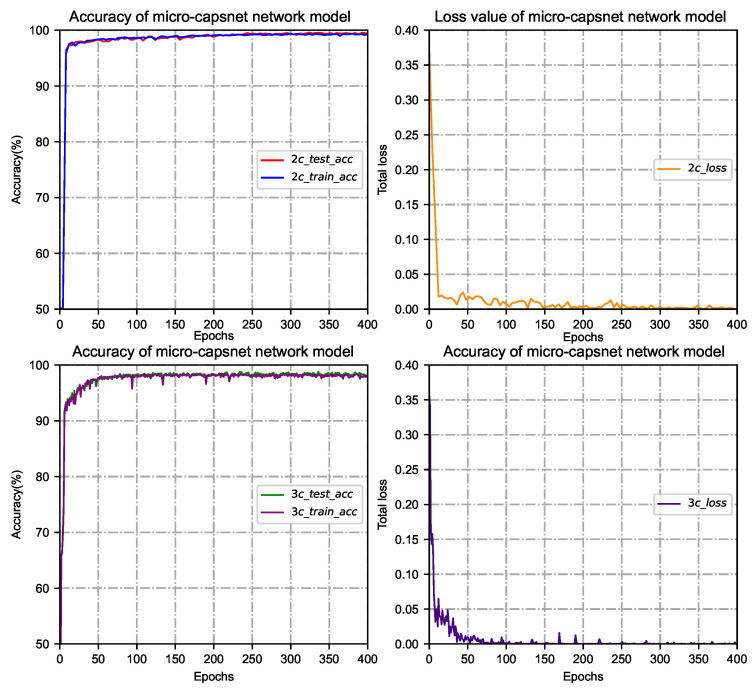
The accuracy and loss graphs for binary and three classifications of training and testing seizure detection.

Since the iterations are an essential parameter of the micro-capsule network model, their value can be used to evaluate the model’s performance. After routing iterative calculation, a set of routing coefficients can be obtained so that the output from the low-level capsule can best match the production of a high-level capsule. In the case of binary classification (such as AB vs. E), three classifications (for example, A vs. C vs. E) of training and testing epileptic detection, the training and testing phases are set to 1000 cycles. After extensive training, testing and operation on different types of epileptic EEG datasets, most of the model training and testing can achieve stable classification results after 400 cycles. The accuracy and loss value of the micro-capsule network model for seizure detection are shown in Figure 3.

As the number of iterations increases, the epileptic recognition rate of the micro-capsule network model increases while the loss value decreases. When the number of iterations reaches 200∼400, the recognition rate and loss in the training phase change slowly and gradually stabilize. At this time, the epileptic recognition rate maintains at about 99%, and the gradient of the loss rate converges smoothly, indicating that the model has a good fit. Considering that the network model is overfitted or takes too long, the number of routing iterations is set to 400 to meet the best match between the output of low-level capsules and that of high-level capsules.

### 3.2. Influence of Time Interval on the Micro-Capsule Networks

**Figure 4:**
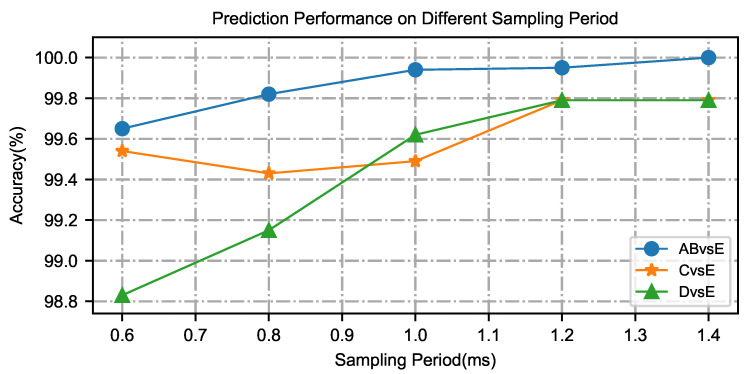
The change trend of recognition rate for EEG segment over different time intervals.

To investigate the influence of individual sample size on the epileptic model during the identification of epilepsy EEG signals, the epilepsy state can be identified in a short time, which can save the identification time and detect epilepsy symptoms as early as possible. Epilepsy EEG signals corresponding to different time intervals are used to detect epilepsy and evaluate the performance of the micro-capsule networks. To obtain the best time interval, the time intervals are separately set to 0.6 s, 0.8 s, 1.0 s, 1.2 s, and 1.4 s, and the correct rate of epilepsy at different time intervals can be obtained through the micro-capsule networks. Taking binary classifications as an example, the changing trend of recognition rate is shown in Figure 4.

**Figure 5:**
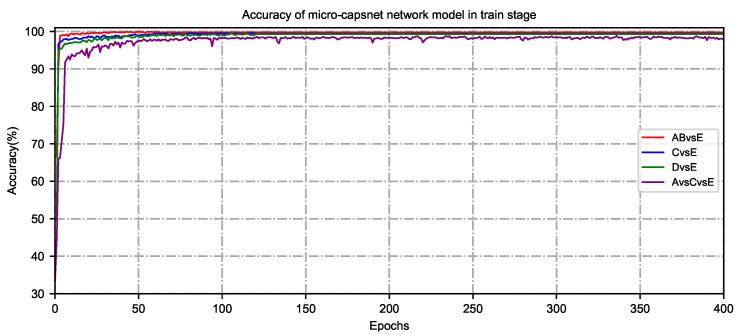
The training iteration times and accuracy of the micro-capsule networks for different dataset cases of EEG epileptic detection.

With the increase in the time interval, the accuracy of epileptic recognition by the micro-capsule networks is gradually improved. That is, when the time intervals are between 0.6 s and 1.4 s, increasing at 0.2 s intervals, the correct rate of epileptic recognition increases steadily. However, after the time intervals are set to greater than 1.0 s, the curve of epilepsy identification correct rate does not increase significantly. Even if the subsequent time length increases, the epilepsy recognition rate only increases by about 0.35%. To recognize epileptic symptoms using a micro-capsule network model and in the shortest possible time, the input time length of epileptic EEG signals is set to 1.0 s, and the training iteration and accuracy of the micro-capsule network model are obtained for different dataset cases, which are AB vs. E, C or D vs. E and A vs. C vs. E., as shown in Figure 5. The experimental results show that the time interval parameter not only ensures the recognition ability of the proposed model but also reduces the epileptic recognition time.

### 3.3. Performance of the Micro-Capsule Network Model

To further evaluate the performance of the micro-capsule network model, the results for EEG signals corresponding to the normal state, inter-ictal and ictal state are calculated with evaluation metrics. Each group of experiments lists the average and standard deviation through paired EEG dataset cases (for example, AB vs. E denotes normal state vs. ictal, C vs. E and D vs. E stand for inter-ictal vs. ictal state, respectively, A vs. C vs. E represents normal state vs. inter-ictal vs. ictal state) under different epileptic states, and the performance results are shown in Table 2.

It can be seen from Table 2 that the accuracy of the proposed model for paired EEG dataset cases are 99.94±0.07%, 99.49±0.41%, 99.62±0.02%, and 99.06±0.22%, respectively. Similarly, the indicators of sensitivity and specificity have a corresponding display. The micro-capsule network model can correctly and stably detect whether it is the binary classifications of normal state and epilepsy state or the three classifications of the normal state, inter-ictal, and ictal state. The results show that the proposed method has better performance of the micro-capsule network model through paired EEG dataset cases for epileptic recognition ability, an average recognition accuracy higher than 99.00%, and good robustness in the case of a small dataset. It can be seen that the proposed framework model has better classification accuracy, which further verifies the effectiveness of this research method.

## 4. Discussion

### 4.1. Advantages of the Micro-Capsule Network Model

Addressing the challenges of small sample sizes, limited individual sample sizes, complex neural network architectures, and subtle epileptic features, this study proposes a micro-capsule network model for epilepsy recognition tasks. Compared with existing capsule network models, the proposed approach demonstrates superior performance for small-sample epilepsy classification, particularly on datasets with limited sample sizes. Results indicate that the model significantly improves performance when applied to small-sample epileptic data. Traditional neural networks for epileptic detection employ deeper architectures to achieve higher recognition rates, which increases computational complexity. In contrast, the novel micro-capsule-based method offers distinct advantages:

The proposed method eliminates the need to reconstruct epileptic EEG signals in the capsule network layer. Additionally, its fully connected structure minimizes network connections, reducing both layer count and architectural complexity.

To enhance feature extraction, the method leverages dimensionality enhancement techniques for EEG signal preprocessing. Combined with convolutional neural network layers, it preserves spatial relationships in subtle epileptic EEG signals, improving feature quality.

By representing EEG features as local signal vectors and employing dynamic routing with fewer training parameters, the model reduces overfitting and accelerates neural network convergence.

### 4.2. Comparisons with Previous Methods

A wide range of algorithms have been proposed in the literature for the automatic detection of epileptic seizures, leveraging the Bonn EEG database. Many studies categorize two or three distinct states, such as normal, inter-ictal, and ictal states. The proposed method is evaluated against existing approaches on the same benchmark dataset, as demonstrated in Table 3.

As can be seen by the above results, the proposed method outperforms most comparative methods. For binary classification problems, classification of healthy volunteers and seizures is done using different dataset combinations from A and B with E (AB vs. E). Zhao et al. proposed a novel 1D-CNN for robust detection of seizures, which could reach 99.38% recognition accuracy. Sharmila and Geethanjali proposed the framework based on a discrete wavelet transform (DWT) analysis and KNN, which achieved an accuracy of 99.16%. The method of epileptic detection with Neo-Natal EEG signals was proposed based on the LSTM classifier, which could reach 99.17% recognition accuracy. The proposed model was recognized with an accuracy of 99.94%. In particular, both sets D and E are difficult to distinguish because the two datasets are from the epileptogenic zone. The recognition rate of Zhao’s method of 1D-CNN could reach 98.02% accuracy. Bhattacharyya et al. achieved 99.50% recognition accuracy with tunable-Q wavelet transform (TQWT) and SVM. Tao Zhang et al. proposed a novel epileptic recognition method combining wavelet packet decomposition (WPD) and KNN, and the highest mean accuracy of 99.36% was obtained. The epileptic recognition rate of the proposed method could achieve 99.49%. Similarly, it is difficult to detect epilepsy in sets D and E from the epileptogenic zone. The epileptic recognition rate of Zhao’s, Bhattacharyya’s, and Tao Zhang’s studies can reach 97.63%, 98.00%, and 98.36%, respectively. The recognition rate of 99.62% is attained by using the proposed method.

For the three-category problem, the recognition rate of Zhao’s method can be up to 96.73%. Behara et al. proposed a novel method with a least-squares support vector machine, and the epileptic recognition accuracy showed a mean accuracy of 97.19%. Türk et al. proposed a new convolutional neural network structure used to learn the properties of these scalogram images, and the classification performance could reach 97.00%. The epilepsy detection method with Neo-Natal EEG was proposed based on the LSTM classifier, which could get 94.81% recognition accuracy. Our proposed model achieved a mean accuracy of 99.06%.

The recognition rate for dichotomous and trichotomous epileptic types is approximately higher than existing methods, which effectively improves the recognition rate of epileptic detection. In addition to the difference in the correct rate of epileptic detection, there is a gradual trend of smaller segments of input epileptic EEG data from traditional machine learning to deep learning algorithms [36,43,44,45,46], and the time of input data segment is set to 1 s, which is worse than most deep learning epileptic detection algorithms. Even if the recognition accuracy of the proposed model is individually smaller than existing algorithms, it does come close to the highest-performing classifier without using hand-crafted features, and it outperforms most other comparative methods. In addition, clinicians have endorsed the epilepsy detection method and associated research outcomes [47,48]. Overall, the micro-capsule network framework can effectively enhance the weakly epileptic EEG features and improve the classification effect in epileptic states by reducing the capsule network layer, which can solve the problem of a small amount of seizure data in clinical applications to some extent. This facilitates real-time detection of epilepsy patients and further prompts patients, their families, and doctors to take necessary preventive measures.

### 4.3. Limitations and Future Works

Although the automatic detection of epileptic EEG signals using the micro-capsule network framework has achieved promising results, the proposed method still has limitations. First, deep learning relies heavily on large quantities of epileptic samples. In practical applications, collecting such samples poses significant risks to patients and imposes substantial burdens on clinicians during data acquisition. Second, existing public epileptic EEG datasets are fragmented and inconsistent with the requirements of real-time seizure-type detection, necessitating refinement of the network framework to support real-time analysis. Third, the proposed framework simplifies the model architecture to optimize computing resources, enable deployment on portable devices, and achieve a more practical design. Fourth, training the micro-capsule network framework for epilepsy EEG signals remains computationally intensive, necessitating further optimization of the architecture and hyperparameters. Additionally, the next step is to develop an independent epilepsy detection application based on capsule neural networks. This application prioritizes a user-friendly interface, enabling clinical doctors to readily access EEG signal analysis results and seizure risk assessments, thereby enhancing interaction with the system.

## 5. Conclusions

Seizures cause physical and mental health disorders, and early detection of epileptic episodes and timely intervention can reduce patient suffering. Many studies have employed machine learning and deep learning methods for seizure detection. In this study, we propose a novel miniaturized capsule network framework to classify epileptic EEG signals. Its innovation lies in the compact capsule network architecture, which preserves the spatial characteristics of subtle epileptic EEG signals while reducing the number of network layers and simplifying the overall design. This study evaluates the efficacy of using the proposed micro-capsule network framework, identifying epileptic EEG signals in small-sample datasets. Experimental results demonstrate that the compact capsule network significantly enhances epilepsy detection, achieving recognition rates of 99.94% (AB vs. E), 99.49% (C vs. E), 99.62% (D vs. E), and 99.06% (A vs. C vs. E). Comparative analysis with existing literature confirms the method’s effectiveness in automated seizure detection. These findings could extend to real-time seizure detection systems, mitigating the duration and discomfort of seizures while reducing clinicians’ workloads. Further development of a lightweight capsule network framework is required to enhance method practicality, specifically addressing real-time processing constraints for single-channel epilepsy EEG signals under limited sample conditions, non-invasive dry or semi-dry electrodes are used to collect EEG signals, thereby expediting clinical translation of detection algorithms.

## Figures and Tables

**Table 1:** The layers and structural parameters of the micro-capsule network model.

Name	Input Shape	Output Shape	Convolution Kernel
Input	(1, 342)	(19, 18, 1)	-
Con-1	(19, 18, 1)	(19, 18, 8)	(4, 4)
Con-2	(19, 18, 8)	(13, 12, 8)	(4, 4)
Primary Capsule	(13, 12, 8)	(312, 4, 1)	(4, 4), (4, 4), (2, 4, 16)
Label Capsule	(312, 4, 1)	(2, 16, 1)	-
Output	(2, 16, 1)	(1, 2)	-

Hyperparameters of the micro-capsule network model employ three setting parameters: optimizer = Adam, learning rate = 0.0005, batch size = 32.

**Table 2:** The performance of the micro-capsule network model through paired EEG dataset cases for epileptic detection.

Data Category	Acc	Spec	Sen
AB vs. E	99.94±0.07%	99.86±0.14%	99.98±0.04%
C vs. E	99.49±0.41%	99.62±0.36%	99.67±0.22%
D vs. E	99.62±0.02%	99.53±0.16%	99.71±0.02%
A vs. C vs. E	99.06±0.22%	98.59±0.003%	98.59±0.003%

**Table 3:** Comparison between the proposed method and existing methods using the same datasets.

Data Category	Methods	Auther	Acc (%)	Our Acc (%)
AB vs. E	1D - CNN	Zhao et al. [36]	99.38%	99.94%
	DWT + KNN	Sharmila et al. [37]	99.16%	
	LSTM	Abbasi et al. [38]	99.17%	
C vs. E	1D - CNN	Zhao et al. [36]	98.02%	99.49%
	TQWT + SVM	Bhattacharyya et al. [39]	99.50%	
	WPD + KNN	Zhang et al. [40]	99.36%	
D vs. E	1D - CNN	Zhao et al. [36]	97.63%	99.62%
	CNN + SVM	Bhattacharyya et al. [39]	98.00%	
	WPD + KNN	Zhang et al. [40]	98.36%	
A vs. C vs. E	1D - CNN	Zhao et al. [36]	96.73%	99.06%
	LSSVM	Behara et al. [41]	97.19%	
	CWT + CNN	Türk and Ömer [42]	97.00%	
	LSTM	Abbasi et al. [38]	97.00%	

## Data Availability

The datasets analyzed for this study can be found in the Bonn University EEG dataset by visiting the following link: https://github.com/RYH2077/EEG-Epilepsy-Datasets (accessed on 25 August 2023).

## References

[1] World Health Organisation (2025). Epilepsy, Website. https://www.who.int/news-room/fact-sheets/detail/epilepsy/.

[2] Zheng G., Kong H. (2024). Exploring the correlation between serum *α*-synuclein and abnormal electroencephalography patterns in children with epilepsy, as well as electroencephalographic discharge index. Int. J. Neurosci..

[3] Cardeña E., Pick S., Litwin R. (2020). Differentiating psychogenic nonepileptic from epileptic seizures: A mixed-methods, content analysis study. Epilepsy Behav..

[4] Liu X., Zhang Y., Zhao Y., Zhang Q., Han F. (2024). The neurovascular unit dysfunction in the molecular mechanisms of epileptogenesis and targeted therapy. Neurosci. Bull..

[5] Kanner A.M., Bicchi M.M. (2022). Antiseizure Medications for Adults with Epilepsy: A Review. JAMA.

[6] Woodbright M., Verma B., Haidar A. (2021). Autonomous deep feature extraction based method for epileptic EEG brain seizure classification. Neurocomputing.

[7] Dutta K.K., Manohar P., Indira K., Naaz F., Lakshminarayan M., Rajagopalan S. (2023). Seven Epileptic Seizure Type Classification in Pre-Ictal, Ictal and Inter-Ictal Stages using Machine Learning Techniques. Adv. Mach. Learn. Artif. Intell..

[8] Wei X., Zhou L., Chen Z., Zhang L., Zhou Y. (2018). Automatic seizure detection using three-dimensional CNN based on multi-channel EEG. BMC Med. Inform. Decis. Mak..

[9] Gupta S., Bagga S., Maheshkar V., Bhatia M. Detection of Epileptic Seizures using EEG Signals. Proceedings of the 2020 International Conference on Artificial Intelligence and Signal Processing (AISP).

[10] Wong S., Simmons A., Rivera-Villicana J., Barnett S., Sivathamboo S., Perucca P., Kwan P., Kuhlmann L., Vasa R., O’Brien T.J. (2023). EEG based automated seizure detection–A survey of medical professionals. Epilepsy Behav..

[11] Shoeibi A., Khodatars M., Ghassemi N., Jafari M., Moridian P., Alizadehsani R., Panahiazar M., Khozeimeh F., Zare A., Hosseini-Nejad H. (2021). Epileptic seizures detection using deep learning techniques: A review. Int. J. Environ. Res. Public Health.

[12] Duan L., Lian Z., Chen J., Qiao Y., Miao J., Li M. (2021). Classification of epilepsy period based on combination feature extraction methods and spiking swarm intelligent optimization algorithm. Concurr. Comput. Pract. Exp..

[13] Moctezuma L.A., Molinas M. (2020). EEG channel-selection method for epileptic-seizure classification based on multi-objective optimization. Front. Neurosci..

[14] Omar A., Abd El-Hafeez T. (2024). Optimizing epileptic seizure recognition performance with feature scaling and dropout layers. Neural Comput. Appl..

[15] Gemein L.A., Schirrmeister R.T., Chrabąszcz P., Wilson D., Boedecker J., Schulze-Bonhage A., Hutter F., Ball T. (2020). Machine-learning-based diagnostics of EEG pathology. NeuroImage.

[16] Kim K., Duc N.T., Choi M., Lee B. (2021). EEG microstate features for schizophrenia classification. PLoS ONE.

[17] Usman S.M., Khalid S., Aslam M.H. (2020). Epileptic seizures prediction using deep learning techniques. IEEE Access.

[18] Bomela W., Wang S., Chou C.A., Li J.S. (2020). Real-time inference and detection of disruptive EEG networks for epileptic seizures. Sci. Rep..

[19] Si Y. (2020). Machine learning applications for electroencephalograph signals in epilepsy: A quick review. Acta Epileptol..

[20] Hu D., Cao J., Lai X., Wang Y., Wang S., Ding Y. (2020). Epileptic state classification by fusing hand-crafted and deep learning EEG features. IEEE Trans. Circuits Syst. II Express Briefs.

[21] Yang Y., Zhou M., Niu Y., Li C., Cao R., Wang B., Yan P., Ma Y., Xiang J. (2018). Epileptic seizure prediction based on permutation entropy. Front. Comput. Neurosci..

[22] Jana R., Mukherjee I. (2021). Deep learning based efficient epileptic seizure prediction with EEG channel optimization. Biomed. Signal Process. Control.

[23] Zhang H., Liu J., Wang B., Dai J., Lian J., Ke A., Zhao Y., Zhou J., Wang C. (2022). Motion direction prediction through spike timing based on micro Capsnet networks. Sci. China Technol. Sci..

[24] Li C., Zhao Y., Song R., Liu X., Qian R., Chen X. (2023). Patient-Specific Seizure Prediction From Electroencephalogram Signal via Multichannel Feedback Capsule Network. IEEE Trans. Cogn. Dev. Syst..

[25] Kumari N., Anwar S., Bhattacharjee V. (2021). Convolutional Neural Network-Based Visually Evoked EEG Classification Model on MindBigData. Proceedings of Research and Applications in Artificial Intelligence.

[26] Liu X., Xie Q., Lv J., Huang H., Wang W. (2021). P300 event-related potential detection using one-dimensional convolutional capsule networks. Expert Syst. Appl..

[27] Sabour S., Frosst N., Hinton G.E. Dynamic routing between capsules. Proceedings of the 31st Conference on Neural Information Processing Systems (NIPS 2017).

[28] Ha K.W., Jeong J.W. (2019). Motor imagery EEG classification using capsule networks. Sensors.

[29] Toraman S. (2021). Automatic recognition of preictal and interictal EEG signals using 1D-capsule networks. Comput. Electr. Eng..

[30] Wang S., Wang G., Pei G., Yan T. An EEG-based approach for Parkinson’s disease diagnosis using capsule network. Proceedings of the 2022 7th International Conference on Intelligent Computing and Signal Processing (ICSP).

[31] Dan J., Pale U., Amirshahi A., Cappelletti W., Ingolfsson T.M., Wang X., Cossettini A., Bernini A., Benini L., Beniczky S. (2024). SzCORE: Seizure Community Open-Source Research Evaluation framework for the validation of electroencephalography-based automated seizure detection algorithms. Epilepsia.

[32] Andrzejak R.G., Lehnertz K., Mormann F., Rieke C., David P., Elger C.E. (2001). Indications of nonlinear deterministic and finite-dimensional structures in time series of brain electrical activity: Dependence on recording region and brain state. Phys. Rev. E.

[33] Emami A., Kunii N., Matsuo T., Shinozaki T., Kawai K., Takahashi H. (2019). Seizure detection by convolutional neural network-based analysis of scalp electroencephalography plot images. NeuroImage Clin..

[34] Mazzia V., Salvetti F., Chiaberge M. (2021). Efficient-capsnet: Capsule network with self-attention routing. Sci. Rep..

[35] Li C., Wang B., Zhang S., Liu Y., Song R., Cheng J., Chen X. (2022). Emotion recognition from EEG based on multi-task learning with capsule network and attention mechanism. Comput. Biol. Med..

[36] Zhao W., Zhao W., Wang W., Jiang X., Zhang X., Peng Y., Zhang B., Zhang G. (2020). A Novel Deep Neural Network for Robust Detection of Seizures Using EEG Signals. Comput. Math. Methods Med..

[37] Sharmila A., Geethanjali P. (2016). DWT based detection of epileptic seizure from EEG signals using naive Bayes and k-NN classifiers. IEEE Access.

[38] Abbasi M.U., Rashad A., Basalamah A., Tariq M. (2019). Detection of epilepsy seizures in neo-natal EEG using LSTM architecture. IEEE Access.

[39] Bhattacharyya A., Pachori R.B., Upadhyay A., Acharya U.R. (2017). Tunable-Q wavelet transform based multiscale entropy measure for automated classification of epileptic EEG signals. Appl. Sci..

[40] Zhang T., Chen W., Li M. (2018). Fuzzy distribution entropy and its application in automated seizure detection technique. Biomed. Signal Process. Control.

[41] Behara D.S.T., Kumar A., Swami P., Panigrahi B.K., Gandhi T.K. Detection of epileptic seizure patterns in EEG through fragmented feature extraction. Proceedings of the 2016 3rd International Conference on Computing for Sustainable Global Development (INDIACom).

[42] Türk Ö., Özerdem M.S. (2019). Epilepsy detection by using scalogram based convolutional neural network from EEG signals. Brain Sci..

[43] Lin L.C., Chen C.J., Chiang C.T., Wu H.C., Yang R.C., Ouyang C.S. (2017). Classification Preictal and Interictal Stages via Integrating Interchannel and Time-Domain Analysis of EEG Features. Clin. EEG Neurosci..

[44] Ma M., Cheng Y., Wei X., Chen Z., Zhou Y. (2021). Research on epileptic EEG recognition based on improved residual networks of 1-D CNN and indRNN. BMC Med. Inform. Decis. Mak..

[45] Billeci L., Tonacci A., Varanini M., Detti P., Vatti G. Epileptic seizures prediction based on the combination of EEG and ECG for the application in a wearable device. Proceedings of the 2019 IEEE 23rd International Symposium on Consumer Technologies (ISCT).

[46] Pachori R.B., Patidar S. (2014). Epileptic seizure classification in EEG signals using second-order difference plot of intrinsic mode functions. Comput. Methods Programs Biomed.

[47] Wong S., Simmons A., Rivera-Villicana J., Barnett S., Sivathamboo S., Perucca P., Ge Z., Kwan P., Kuhlmann L., Vasa R. (2023). EEG datasets for seizure detection and prediction—A review. Epilepsia Open.

[48] Chen W., Wang Y., Ren Y., Jiang H., Du G., Zhang J., Li J. (2023). An automated detection of epileptic seizures EEG using CNN classifier based on feature fusion with high accuracy. BMC Med. Inform. Decis. Mak..

